# Isoniazid-Associated Serotonin Toxicity in the Critical Care Setting: A Case Report

**DOI:** 10.7759/cureus.82362

**Published:** 2025-04-16

**Authors:** Matthew Gunther, Jordan Broadway, Jose R Maldonado

**Affiliations:** 1 Psychiatry and the Behavioral Sciences, Stanford University School of Medicine, Palo Alto, USA; 2 Psychiatry, University of Southern California, Los Angeles, USA

**Keywords:** drug drug interactions, isoniazid, mao inhibitors, serotonin syndrome (ss), serotonin toxicity

## Abstract

Serotonin syndrome presents with the triad of neuromuscular excitability, autonomic disturbance, and altered mental status, resulting from excess serotonergic tone. Isoniazid (INH), a core agent for the management of tuberculosis (TB), is a weak, non-selective monoamine oxidase inhibitor (MAOI), and there have been minimal reports of its potential to contribute to serotonin toxicity. We present a complex case of INH-associated serotonin toxicity in a patient with autism spectrum disorder and co-occurring severe TB in the critical care setting. The patient was on rifampin, INH, pyrazinamide, and ethambutol regimen (RIPE) for pulmonary TB. Due to severe, refractory agitation for over a week, which prevented weaning of sedation and extubation, psychiatry was consulted. The psychiatry team worked to address agitation through a combination of haloperidol, lithium, valproic acid (VPA), and pregabalin. The patient developed serotonin toxicity, which persisted despite the cessation of psychotropics with serotonergic potential. This report illustrates the potential of INH’s MAO inhibition to contribute to the development of serotonin toxicity. Consulting psychiatrists should exercise caution when recommending psychotropics to patients receiving INH and should take into account pharmacodynamic and pharmacokinetic interactions associated with its use. We recommend regular screening for serotonin toxicity in patients on INH and other agents that can increase serotonergic serum levels.

## Introduction

Serotonin syndrome manifests with the triad of neuromuscular excitability, autonomic disturbance, and altered mental status secondary to excess serotonergic tone. Its symptoms can range in severity (with the term “serotonin toxicity” better representing this spectrum diagnosis), usually develops with the administration of two or more serotonergic agents, and is dose-dependent. Monoamine oxidase inhibitors (MAOIs) are often implicated in this condition; this class of medications prevents the breakdown of several neurotransmitters, including serotonin, norepinephrine, and dopamine. Linezolid, a weak, non-selective inhibitor of monoamine oxidase used for resistant infections in critical care settings, is commonly referenced in literature as a non-psychotropic medication associated with the development of serotonin toxicity (although recent data indicate that the incidence is low) [1]. Isoniazid (INH), a core agent in the management of tuberculosis (TB), is also a weak, non-selective MAOI, although reports of its potential to contribute to serotonin toxicity are minimal [2,3]. In this case report, we describe a complex case of INH-associated serotonin toxicity in the critical care setting.

## Case presentation

The patient was a 23-year-old female with autism spectrum disorder who presented with hemoptysis, fatigue, and weight loss. At baseline, the patient was described as non-verbal and dependent on all activities of daily living; her primary caregivers were her parents. She was intubated upon presentation and admitted to intensive care for acute hypoxic respiratory failure. Workup revealed multiple cavitary lesions bilaterally, and bronchoscopy was consistent with severe pulmonary TB. The patient was started on a rifampin, INH, pyrazinamide, and ethambutol regimen (RIPE). On week three of hospitalization, psychiatry was consulted to manage severe agitation (thrashing, pulling at lines, physical aggression with nursing staff) for over one week, preventing weaning of sedation and extubation.

On initial evaluation, the patient was sedated on dexmedetomidine, propofol, and hydromorphone, with standing guanfacine (6 mg/day), olanzapine 30 mg/day, and lorazepam (18 mg/day). Attempts at reducing sedation led to physical agitation and autonomic excitability. An interview was not very fruitful, and the exam was unremarkable. The working diagnosis at the time was delirium due to critical illness, prolonged ICU stay, and agent selection for sedation (including benzodiazepines and opioids, which can lead to/exacerbate delirium) - although a complete neuropsychiatric exam was not possible given the patient's baseline communication limitations. Over three weeks, psychiatry facilitated weaning sedation to lower levels through the addition of valproic acid (VPA) (2 g/day; level of 68 mcg/mL), conversion of olanzapine to intravenous haloperidol (15 mg/day), initiation of pregabalin for agitation/pain (45 0mg/day), and ramelteon/daridorexant for sleep. Lorazepam was slowly reduced to 6 mg/day over three weeks.

On week six, the psychiatry team elicited upper extremity rigidity and cogwheeling without clonus, attributed to extrapyramidal symptoms from haloperidol. To taper this agent and target residual agitation, lithium was added to manage agitation (ongoing thrashing and hitting nursing staff when routine care was provided) and titrated to 600 mg/day (serum level: 0.5 mEq/L). On week eight, lithium was increased to 900 mg/day for additional behavioral control (including ongoing physical aggression with staff and intermittent pulling at lines). The next day, the patient developed both spontaneous and inducible clonus throughout the upper and lower extremities, diaphoresis, tachycardia, and diarrhea. Given that the patient demonstrated neuromuscular excitability (not hypokinesis or lead-pipe rigidity) and was not exposed to dopamine antagonists for two weeks, neuroleptic malignant syndrome was ruled out. Lithium toxicity was briefly considered, but an increase from 0.5 mEq/L to a toxic level was not expected with a 300 mg increase the day before (and the level was later noted to be 0.65 mEq/L).

The patient was eventually diagnosed with serotonin toxicity based on Hunter criteria, precipitated by a combination of INH and her psychotropics (as above: lithium, VPA, and pregabalin; additional as needed oxycodone up to 15 mg/day for agitation thought to be due to pain). Lithium was held, and cyproheptadine was initiated as antidotal therapy. Pregabalin and VPA were discontinued; INH was maintained, given severe TB, and low-dose opioids (oxycodone 2.5-7.5 mg/day) were maintained for pain control. Despite adjustments and further care, the patient's condition continued to worsen clinically, and she continued to demonstrate signs of serotonin toxicity, including autonomic instability, diaphoresis, and clonus (thought to be perpetuated by continued INH use with low-dose opioids). Throughout this time, the psychiatry team participated in family meetings (along with the primary team and palliative care) surrounding the goals of care, given the patient's tenuous respiratory status. After several goals-of-care meetings, at which point her pulmonary status continued to worsen despite aggressive intervention (including INH), the patient was transferred to comfort care and subsequently passed away.

## Discussion

Serotonin toxicity was first described in patients taking MAOI and L-tryptophan, but it is currently associated with a range of serotonergic medications, including antidepressants, opioids, and antimicrobial agents [4-6]. As a class, MAOIs vary in selectivity and reversibility, with serotonin toxicity associated with more potent, irreversible inhibitors (such as tranylcypromine). Serotonin toxicity develops when net serotonergic tone is high; even mild inhibition of MAO (INH is a weak, reversible inhibitor), when combined with other serotonergic agents, can precipitate toxicity [2,4]. This is particularly relevant in critical care settings where polypharmacy is common. A recent study showed that 7.8% of patients developed serotonin syndrome in the ICU [7]. In the study, most patients received two or more serotonergic agents, and non-psychotropics were a major factor in the development of serotonin toxicity [7].

INH is often used as part of quadruple therapy to treat tuberculosis [8]. TB, caused by Mycobacterium tuberculosis, is a highly contagious pulmonary disease with the potential for extrapulmonary involvement. INH acts to disrupt bacterial growth by inhibiting mycolic acid synthesis [8]. INH is a crucial component of treatment, both acutely and in the continuation phase. Its neuropsychiatric adverse effects include headache, insomnia, depression, and cognitive effects, though peripheral neuropathy is most common (up to 20%) [8-9]. Because of its adverse effect profile (including boxed warning for severe hepatotoxicity) and growing INH resistance, experts have explored whether INH is a necessary component of the regimen [8]. In some cases, therapies without INH have been successful; however, experts hold the view that its ongoing use is essential, especially in cases complicated by sepsis or extrapulmonary tuberculosis (given its CNS penetrance) [8].

Consulting psychiatrists are tasked with utilizing their expertise in navigating complex drug interactions, including recognition and mitigation of serotonin toxicity. Pharmacodynamically, INH acts to inhibit CYP3A4 and CYP2C19 enzymes and can elevate VPA levels, although this was not the case with our patient [10]. INH does have MAOi activity, and while the data on its potential to contribute to serotonin toxicity are limited, its essential role in TB treatment complicates traditional psychopharmacologic management [2]. In the ICU setting, psychiatrists may need to utilize multiple pharmacologic agents to address agitation secondary to delirium or distress, as in our case. Many components of the psychiatrist’s pharmacologic armamentarium can increase serotonin; in our case, this included lithium, VPA, and pregabalin [6,11-13]. This case was particularly challenging given these contributors in addition to the patient's underlying critical illness. While the initial diagnosis of serotonin toxicity was made in the setting of multiple serotonergic agents, the syndrome persisted despite reduction/cessation of most of these agents, except for INH. While INH may be considered a weak MAOI (compared to MAOIs used in the treatment of depression), this case highlights INH's potential to both contribute to the development and complicate the management of serotonin toxicity.

## Conclusions

INH remains a staple of TB management. Consulting psychiatrists should exercise caution in recommending psychotropics to patients receiving INH and should consider pharmacodynamic and pharmacokinetic interactions linked to its use, including CYP enzyme inhibition and increased serotonergic tone. Awareness of signs of serotonin toxicity (including differentiating the syndrome from other pharmacologic emergencies) is crucial to consulting in the critical care setting. We advocate regular screening for serotonin toxicity in patients on INH and other agents that can increase serotonergic serum levels when recommending pharmacologic intervention.
